# Supplementary material for: Unifying the global phylogeny and environmental distribution of ammonia-oxidising archaea based on amoA genes
Source: Nat Commun. 2018 Apr 17;9:1517. doi: 10.1038/s41467-018-03861-1 (PMC5904100; doi:10.1038/s41467-018-03861-1)
Supplement: Supplementary file 1 — Supplementary Information [file 41467_2018_3861_MOESM1_ESM.pdf]

Unifying the global phylogeny and environmental distribution of ammonia-oxidising archaea based on *amoA* genes

Alves *et al*.

Supplementary Information

- Supplementary Discussion
- Supplementary Fig. 1 – Schematic display of assembly, curation, and phylogenetic analyses of archaeal *amoA* gene sequences
- Supplementary Fig. 2 – Phylogeny of AOA and TACK superphylum based on 16S-23S rRNA genes
- Supplementary Table 1 – Reference strains and genomes/genes analysed in this study
- Supplementary Table 2 – Summary of *amoA* sequences from SIP experiments included in this study
- Supplementary References

Supplementary Discussion

Evolution of the archaeal AMO within the CuMMO superfamily. All CuMMOs comprise three subunits, A, B and C, with some organisms containing additional putative subunits[1], such as the AmoX of AOA[2]. Phylogeny inference was based on partitioned model analysis of 125 concatenated subunits A and B, following the assumption that these subunits are mutually essential for enzymatic function and thus their combined evolutionary history should reflect that of the enzyme. This was motivated by previous phylogenies, including our own preliminary analysis, which indicated that individual subunits do not contain sufficient information to reliably model basal evolutionary relationships. Subunit C was excluded since it is present in multiple different (although similar) copies in several organisms. Given the high sequence dissimilarity among many CuMMO proteins, sequences were aligned after consensus-based analysis of 14 independent protein alignment methods and then back-translated for codon-based analyses.

The codon-based phylogeny resolved robust basal relationships between approximate phylum-level lineages, reflected by two interconnected trifurcations: one shared by archaeal and actinobacterial CuMMO lineages, and another shared by verrucomicrobial particulate methane monooxygenases (pMMOs) and the lineage that radiated into *Alpha*-, *Beta*- and *Gammaproteobacteria*, division NC10 and Nitrospirae. Previous phylogenies of individual CuMMO subunits could not reliably resolve this basal topology likely due to insufficient phylogenetic information encoded by the (partial) protein sequences of individual subunits used[3–9]. Moreover, earlier phylogenies also did not simultaneously include all CuMMO diversity included here, some of which was unknown at the time. In turn, our evolutionary model tests showed that codon models fit the CuMMO data better than protein models, in line with previous evidence that codon models accounting for synonymous substitutions improve phylogenetic inference of highly divergent proteins[10], such as those analysed here.

The CuMMO phylogeny indicates five possible scenarios for the origin of archaeal AMOs (Fig. 10): (i) archaeal AMOs, actinobacterial hydrocarbon monooxygenases and other bacterial monooxygenases evolved as three independent lineages from a common ancestor; (ii) CuMMOs emerged in Actinobacteria, or from an ancestor at the root of actinobacterial monooxygenases, and archaeal AMOs evolved from a common ancestor with all other bacterial monooxygenases; (iii) CuMMOs emerged within Bacteria other than Actinobacteria, and archaeal AMOs evolved from a common ancestor with the latter; (iv) CuMMOs evolved initially in AOA and were later acquired by Bacteria, where they radiated into different phyla; (v) archaeal AMOs and bacterial monooxygenases evolved independently from a common ancestral enzyme. It should nevertheless be noted that such distant evolutionary relationships, as that reflected by the long branch of archaeal AMOs, are prone to generate artefacts despite apparent robustness of the trees, even when potential technical bias are extensively accounted for, as done here.

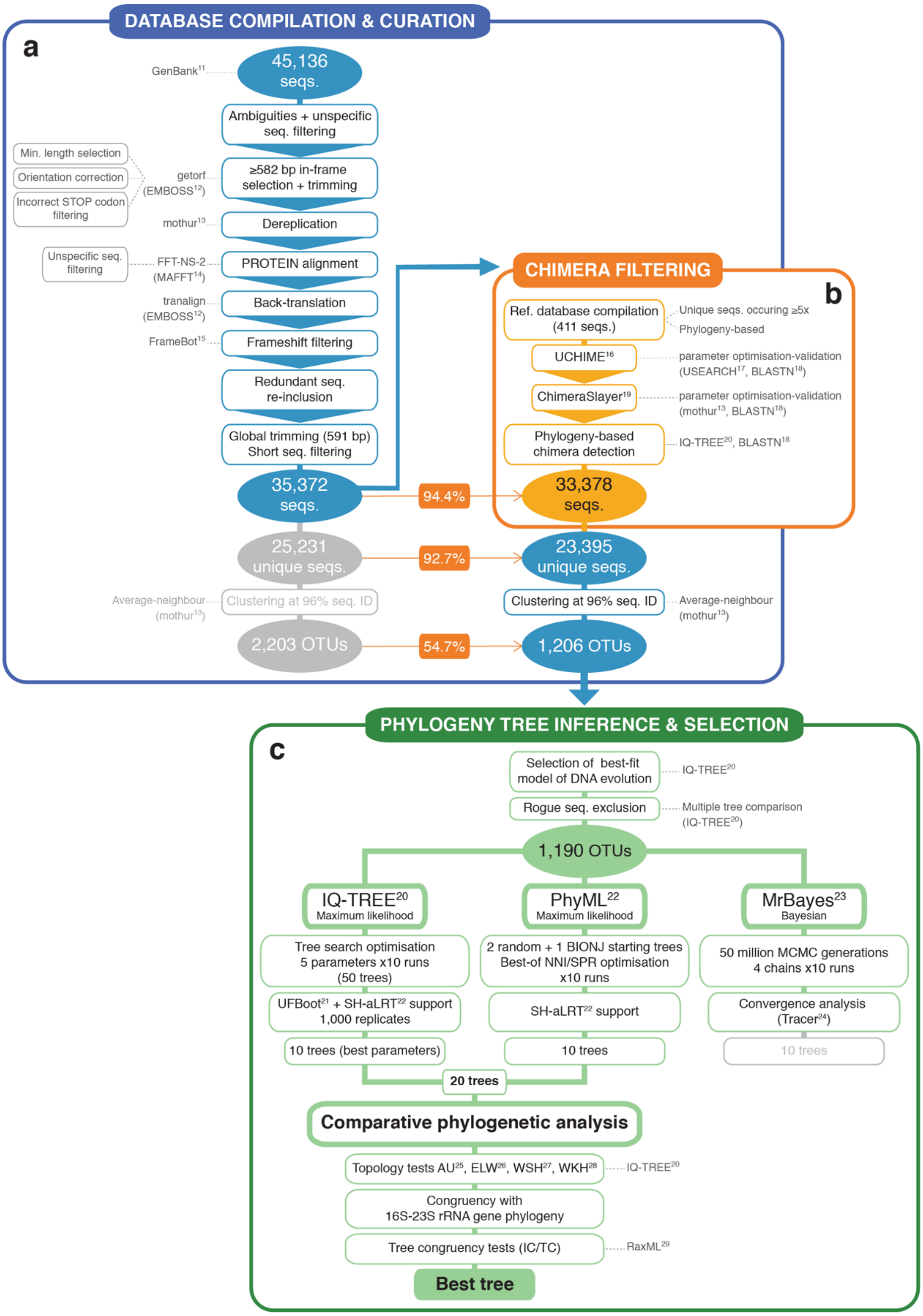

Supplementary Fig. 1. Schematic display of assembly, curation, and phylogenetic analyses of archaeal *amoA* gene sequences. **a)** Primary sequence selection and curation, alignment and trimming (blue). **b)** Chimera filtering after compilation and validation of the reference database for chimera detection (orange); percentage values in orange boxes represent the fraction of sequences in the respective datasets after chimera exclusion. **c)** Optimisation of phylogenetic tree inference and selection of the best tree based on comparative phylogenetic methods (green). Abbreviations: Seq., sequence(s); ID, identity.

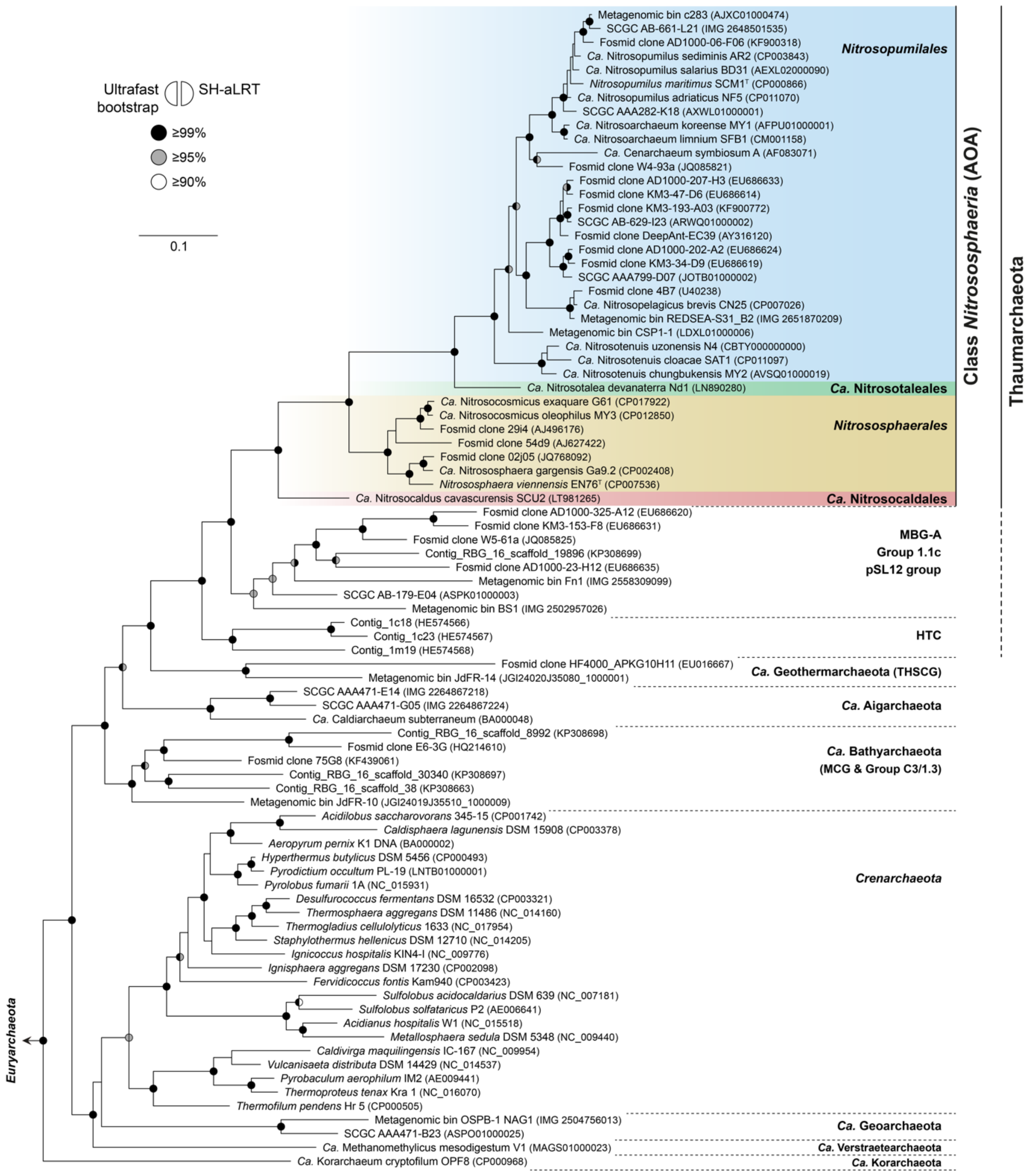

Supplementary Fig. 2. Phylogeny of AOA and TACK superphylum based on 16S-23S rRNA genes. Uncollapsed display of the tree shown in Fig. 2. The tree was inferred from 92 concatenated 16S and 23S rRNA genes (4,628 aligned positions), including representatives of known Thaumarchaeota and TACK candidate phyla, representatives of all Crenarchaeota genera, and representatives of eight Euryarchaeota classes (outgroup). Sequence alignments were based on archaea-specific structurally-accurate seed alignments. The tree was inferred by maximum likelihood with IQ-TREE[20] based on the model GTR+F+R6 with gene partitions using an edge-unlinked partition model[30]. Support values for branches with both ultrafast bootstrap[21] and SH-aLRT[22] >90% (1,000 replicates) are indicated by semi-circles. The scale bar represents substitutions *per* nucleotide position. Sequence accession numbers are provided in Supplementary Table 1. Abbreviations: MBG-A, Marine Benthic Group A; HTC, Hot Thaumarchaeota-related Clade; THSCG, Terrestrial Hot Spring Crenarchaeotic Group; MCG, Miscellaneous Crenarchaeotic Group.

Supplementary Table 1. Reference strains and genomes/genes analysed in this study.

Taxonomy*				Organism/genome	Accession ID	Protein-coding genes (No)[†]
Order	Family	Genus	*amoA*-based			
Nitrosopumilales	*n. d.*	*n. d.*	NP-α-2.2.2.1.1.1	Metagenomic bin REDSEA-S27_B13N2	IMG 2654122917	1973
			n. d.	SCGC AAA799-D07	JOTB01000002	-
			n. d.	Fosmid clone KM3-34-D9	EU686619	-
			n. d.	Fosmid clone AD1000-202-A2	EU686624	-
			NP-α-2.2.2.1.2.1	SCGC AAA007-O23	ARWO01000005	1354
			NP-α-2.2.2.1.2.1	SCGC AB-629-I23	ARWQ01000001	1239
			NP-α-2.2.2.1.2.1	SCGC AC-312-J09	IMG 2667456761	1298
			n. d.	Fosmid clone AD1000-207-H3	EU686633	-
			n. d.	Fosmid clone KM3-47-D6	EU686614	-
			n. d.	Fosmid clone KM3-193-A03	KF900772	-
			n. d.	Fosmid clone DeepAnt-EC39	AY316120	-
	Nitrosopumilaceae	*Nitrosopumilus*	NP-γ-2.1.2	SCGC AAA282-K18	AXWL01000027	1249
			NP-γ-2.1.3	*Ca.* Nitrosopumilus sp. NAOA2	KT380499	-
			NP-γ-2.1.3.1	*Nitrosopumilus cobalaminigenes* HCA1^{T}	KF957665	-
			NP-γ-2.1.3.1	*Nitrosopumilus oxyclinae* HCE1^{T}	KX950755	-
			NP-γ-2.1.3.1	SCGC AD-305-F05	IMG 2658561329	1551
			NP-γ-2.1.3.1	SCGC AD-308-D05	IMG 2667451876	1281
			NP-γ-2.1.3.1	SCGC AD-311-O21	IMG 2638762823	1428
			NP-γ-2.1.3.2	*Ca.* Nitrosopumilus adriaticus NF5	CP011070	2206
			NP-γ-2.1.3.2	*Ca.* Nitrosopumilus koreense AR1[‡]	CP003842	1890
			NP-γ-2.1.3.2	*Ca.* Nitrosopumilus sediminis AR2	CP003843	1974
			NP-γ-2.1.3.2.1	*Nitrosopumilus maritimus* SCM1^{T}	CP000866	1797
			NP-γ-2.1.3.2.1	*Ca.* Nitrosopumilus piranensis D3C	CP010868	2140
			NP-γ-2.1.3.2.1	*Ca.* Nitrosopumilus sp. NAOA6	KT380500	-
			NP-γ-2.1.3.2.1	SCGC RSA3	JOTD01000072	2407
			NP-γ-2.1.3.2.1	SCGC AAA799-D11	JOSY01000059	1733
			NP-γ-2.1.3.2.1	SCGC AAA799-N04	JOKN01000008	1767
			NP-γ-2.1.3.2.3.2	*Ca.* Nitrosopumilus salarius BD31[‡]	AEXL00000000	2089
			NP-γ-2.1.3.2.3.2	*Ca.* Nitrosopumilus sp. NM25	AB546962	-
			NP-γ-2.1.3.2.3.2	*Nitrosopumilus ureiphilus* PS0^{T}	KF957666	-
			n. d.	SCGC AB-661-L21	2648501535	-
			n. d.	Metagenomic bin c283	AJXC01000474	-
			n. d.	Fosmid clone AD1000-06-F06	KF900318	-
		Ca. Nitrosoarchaeum	NP-γ-2.2.5.1	*Ca.* Nitrosoarchaeum koreense MY1[‡]	AFPU01000001	1945
			NP-γ-2.2.5.1	*Ca.* Nitrosoarchaeum sp. AOA-AC2	JQ669392	-
			NP-γ-2.2.5.1	*Ca.* Nitrosoarchaeum limnium SFB1[‡]	CM001158	2038
			NP-γ-2.2.5.1	*Ca.* Nitrosoarchaeum limnium BG20[‡]	NZ_AHJG01000287	2639
		Ca. Cenarchaeum	NP-γ-1.1	*Ca.* Cenarchaeum symbiosum A	DP000238	2017
		n. d.	*n. d.*	Fosmid clone W4-93a	JQ085821	-
	n. d.	*n. d.*	NP-δ-2.1.2	Metagenomic bin CSP1-1	LDXL01000004	1652
	Ca. Nitrosopelagicaceae	*Ca.* Nitrosopelagicus	NP-ε-2.2	*Ca.* Nitrosopelagicus brevis CN25	CP007026	1469
			NP-ε-2.1	Metagenomic bin REDSEA-S25_B3	IMG 2654120203	1257
			NP-ε-2.1	Metagenomic bin REDSEA-S31_B2	IMG 2654123780	1346
			NP-ε-2.1	Metagenomic bin REDSEA-S37_B6	IMG 2654125451	1194
			n. d.	Fosmid clone 4B7	U40238	-
	Ca. Nitrosotenuaceae	*Ca.* Nitrosotenuis	NP-η-1.1.1.2	*Ca.* Nitrosotenuis cloacae SAT1	CP011097	1876
			NP-η-1.2.1	*Ca.* Nitrosotenuis chungbukensis MY2	AVSQ01000002	2024
			NP-η-1.2.1	*Ca.* Nitrosotenuis sp. AOA-DW	JQ669394	-
			NP-η-1.2.2.2	*Ca.* Nitrosotenuis uzonensis N4	CBTY000000000	1909
			NP-η-1.2.2.2	*Ca.* Nitrosotenuis sp. AOA-AC5	JQ669393	-
Ca. Nitrosotaleales	*Ca.* Nitrosotaleaceae	*Ca.* Nitrosotalea	NT-α-1.1.1.1.1	*Ca.* Nitrosotalea devanaterra Nd1	LN890280	2103
			NT-α-1.1.1.1.3.2.2	*Ca.* Nitrosotalea sp. Nd2	KJ540206	-
Nitrososphaerales	*Nitrososphaeraceae*	*Nitrososphaera*	NS-α-3.2.1.1.1.1.1.1	*Nitrososphaera viennensis* EN76^{T}	CP007536	3137
			NS-α-3.2.1.1.1.1.1.1	*Ca.* Nitrososphaera evergladensis SR1	CP007174	3505
			NS-α-3.2.1.1.1.1.IS.1	*Ca.* Nitrososphaera sp. JG1	JF748723	-
			NS-α-3.2.4	*Ca.* Nitrososphaera gargensis Ga9.2	CP002408	3562
			n. d.	Fosmid clone 02j05	JQ768092	-
		Ca. Nitrosocosmicus	NS-ζ	*Ca.* Nitrosocosmicus oleophilus MY3	CP012850	3722
			NS-ζ	*Ca.* Nitrosocosmicus arcticus Kfb	KX863714	-
			NS-ζ-2	*Ca.* Nitrosocosmicus exaquare G61	CP017922	3162
			NS-ζ-2	*Ca.* Nitrosocosmicus franklandus C13	KU290366	-
			n. d.	Fosmid clone 29i4	AJ496176	-
	n. d.	*n. d.*	NS-δ-1.1.2.1	Fosmid clone 54d9	AJ627422	-
Ca. Nitrosocaldales	*Ca.* Nitrosocaldaceae	*Ca.* Nitrosocaldus	NC-α	*Ca.* Nitrosocaldus yellowstonensis HL72[‡]	EU239961	-
			NC-α	*Ca.* Nitrosocaldus cavascurensis SCU2	LT981265	-

n. d., not determined

* The taxonomy follows that defined in Kerou *et al.*, *Nitrososphaeria*. in Bergey's Manual of Systematics of Archaea and Bacteria[31]

[†] Protein-coding genes annotated in IMG/M, regardless of genome completeness

[‡] The original strain names were corrected following Kerou *et al.*[31] to comply with the International Code of Nomenclature of Prokaryotes

Supplementary Table 2. Summary of *amoA* sequences from SIP experiments included in this study.

Study	Environment	Original reference*	% of labelled genes†	*amoA*-based taxonomy	Database reference	Database match % ID
Shi *et al.*, 2016[32]	Soil (agricultural)	KT861612	5%	NP-γ-2.2.5.IS.1	DQ148653	97.0%
		KT861611	79%	NP-η-1.2.1	HM637854	97.6%
		KT861615	5%	NS-α-3.2.1.1.1.2	KF053684	98.5%
		KT861614	5%	NS-δ-1.1.2.1	AB688020	98.6%
		KT861613	5%	NS-ε-2.2	KC756646	97.1%
Zhang *et al.*, 2015[33]	Estuarine-coastal (water/sediments)	*KC735443*	70-75%	NP-α-2.2.4	JQ345747	99.8%
		HQ455773	<1%	NP-γ-2.1	HQ455773	100%
		FJ227809	<1%	NP-γ-2.1.3.2	FJ227809	100%
		AB546962	<1%	NP-γ-2.1.3.2.3.2	EU925261	96.4%
		DQ278501	<1%	NP-γ-2.2.2.1	DQ278570	99.8%
		KC735190	<1%	NP-γ-2.2.2.1	KC735190	100%
		KC735358	<5%	NP-γ-2.2.5	GQ331564	96.3%
		HM345610	25-30%	NP-γ-2.2.5.1	AFPU000001	99.3%
Zhao *et al.*, 2015[34]	Soil (agricultural; paddy)	KP890809	98%	NS-α-3.2.1.1.2	AB625946	99.8%
		KP890811	<1%	NS-δ-1.1.2.1	EU590637	97.4%
		KP890812	<1%	NS-ζ-2	KC758445	96.4%
Wang *et al.*, 2015[35]	Soil (agricultural; paddy)	*JQ319452*	20%	NS-α-3.2.1.1.1.2	KF053684	99.8%
		JQ990071	20%	NS-β-1	KJ864239	99.7%
		JQ345901	95%	NS-ζ-2	JQ345901	100%
		JQ345901	60%	NS-ζ-2	JQ345901	100%
		JQ345901	80%	NS-ζ-2	JQ345901	100%
Wang *et al.*, 2014[36]	Soil (acidic)	*EF207213*	100%	NS-α-3.2.1.1.2	AB625946	99.8%
Wu *et al.*, 2013[37]	Freshwater sediment (eutrophic lake)	*HQ538551*	18%	NS-α-3.2.3.1.6.2	KF857040	99.0%
		EU590198	78%	NS-ζ-2	JQ345901	99.8%
Lu & Jia, 2013[38]	Soil (forest)	JX239702	100%	NT-α-1.1.1.1.3.2.IS	FJ517347	99.3%
	Soil (tea orchard)	JX239696	100%	NT-α-1.1.2.2	JF681783	99.8%
Zhang *et al.*, 2012[39]	Soil (acidic)	JF681783	*n. a.*	NT-α-1.1.2.2	JF681783	100%
		JF681784	*n. a.*	NS-γ-2.3.2.1	AB545961	98.0%
		JF681789	*n. a.*	NS-γ-2.3.2.1	FN869066	100%
Pratscher *et al.*, 2011[40]	Soil (agricultural)	HQ685801	94%	NS-α-3.2.1.1.1.2	KF053684	99.7%
		HQ685810	6%	NS-α-3.2.1.1.2	AB625946	97.9%
		HQ685771	90%	NS-β-1	AB353494	99.8%
		HQ685778	10%	NS-β-1	EU671395	98.5%
Xia *et al.*, 2011[41]	Soil (agricultural)	HQ678188	32%	NS-α-3.2.1.1.1.1.IS.1	KF041530	99.2%
		HQ678187	47%	NS-α-3.2.1.1.1.2	KF053684	98.0%
		HQ678193	6%	NS-α-3.2.1.1.1.2	KF803043	99.2%
		HQ678191	3%	NS-α-3.2.3.1.4.1	EU590224	99.5%
		HQ678192	3%	NS-α-3.2.3.1.6.2	KF857040	98.0%
		HQ678189	6%	NS-α-3.2.4	JQ735263	99.5%
		HQ678190	3%	NS-γ-2.1.1	HQ678190	100%
Zhang *et al.*, 2010[42]	Soil (agricultural)	*FJ971891*	11%	NP-γ-2.2.5	HQ168103	99.2%
		FJ971894	89%	NP-η-1.2.1	KF041507	97.6%
Jiang *et al.*, 2015[43]	Soil (agricultural; paddy)	*HM754670*	95%	NS-α-3.2.1.1.1.1.1.2	LK055904	99.0%
		KC537404	5%	NS-ε-2.1	HQ202397	99.0%

Abbreviations: IS, *Incertae sedis*; ID, identity; *n. a.*, not available

* If the original sequence could not be identified, the closest related sequence in the reference phylogeny was used (indicated in italics)

† Percentage of sequences in individual gene sequence libraries generated from SIP heavy fractions

Supplementary References

1. Simon, J. & Klotz, M. G. Diversity and evolution of bioenergetic systems involved in microbial nitrogen compound transformations. *Biochim. Biophys. Acta* **1827**, 114–135 (2013).
2. Bartossek, R., Spang, A., Weidler, G. W., Lanzén, A. & Schleper, C. Metagenomic analysis of ammonia-oxidizing archaea affiliated with the soil group. *Front. Microbiol.* **3**, 208 (2012).
3. Tavormina, P. L., Orphan, V. J., Kalyuzhnaya, M. G., Jetten, M. S. M. & Klotz, M. G. A novel family of functional operons encoding methane/ammonia monooxygenase-related proteins in gammaproteobacterial methanotrophs. *Environ. Microbiol. Rep.* **3**, 91–100 (2011).
4. Sayavedra-Soto, L. A. et al. The membrane-associated monooxygenase in the butane-oxidizing Gram-positive bacterium Nocardioides sp. strain CF8 is a novel member of the AMO/PMO family. *Environ. Microbiol. Rep.* **3**, 390–396 (2011).
5. Coleman, N. V et al. Hydrocarbon monooxygenase in Mycobacterium: recombinant expression of a member of the ammonia monooxygenase superfamily. *ISME J.* **6**, 171–182 (2012).
6. Lawton, T. J., Ham, J., Sun, T. & Rosenzweig, A. C. Structural conservation of the B subunit in the ammonia monooxygenase/particulate methane monooxygenase superfamily. *Proteins Struct. Funct. Bioinforma.* **82**, 2263–2267 (2014).
7. van Kessel, M. A. H. J. et al. Complete nitrification by a single microorganism. *Nature* **528**, 555–559 (2015).
8. Daims, H. et al. Complete nitrification by Nitrospira bacteria. *Nature* **528**, 504–9 (2015).
9. Oswald, K. et al. Crenothrix are major methane consumers in stratified lakes. *ISME J.* **11**, 2124–2140 (2017).
10. Seo, T.-K. & Kishino, H. Synonymous Substitutions Substantially Improve Evolutionary Inference from Highly Diverged Proteins. *Syst. Biol.* **57**, 367–377 (2008).
11. Clark, K., Karsch-Mizrachi, I., Lipman, D. J., Ostell, J. & Sayers, E. W. GenBank. *Nucleic Acids Res.* **44**, D67–D72 (2016).
12. Rice, P., Longden, I. & Bleasby, A. EMBOSS: the European Molecular Biology Open Software Suite. *Trends Genet.* **16**, 276–7 (2000).
13. Schloss, P. D. et al. Introducing mothur: open-source, platform-independent, community-supported software for describing and comparing microbial communities. *Appl. Environ. Microbiol.* **75**, 7537–7541 (2009).
14. Katoh, K. & Standley, D. M. MAFFT multiple sequence alignment software version 7: Improvements in performance and usability. *Mol. Biol. Evol.* **30**, 772–780 (2013).
15. Wang, Q. et al. Ecological Patterns of nifH Genes in Four Terrestrial Climatic Zones Explored with Targeted Metagenomics Using FrameBot, a New Informatics Tool. *MBio* **4**, e00592-13 (2013).
16. Edgar, R. C., Haas, B. J., Clemente, J. C., Quince, C. & Knight, R. UCHIME improves sensitivity and speed of chimera detection. *Bioinformatics* **27**, 2194–2200 (2011).
17. Edgar, R. C. Search and clustering orders of magnitude faster than BLAST. *Bioinformatics* **26**, 2460–2461 (2010).

18. Altschul, S. F. et al. Gapped BLAST and PSI-BLAST: A new generation of protein database search programs. *Nucleic Acids Res.* **25**, 3389–3402 (1997).

19. Haas, B. J. et al. Chimeric 16S rRNA sequence formation and detection in Sanger and 454-pyrosequenced PCR amplicons. *Genome Res.* **21**, 494–504 (2011).

20. Nguyen, L.-T., Schmidt, H. A., von Haeseler, A. & Minh, B. Q. IQ-TREE: A Fast and Effective Stochastic Algorithm for Estimating Maximum-Likelihood Phylogenies. *Mol. Biol. Evol.* **32**, 268–274 (2015).

21. Minh, B. Q., Nguyen, M. A. T. & von Haeseler, A. Ultrafast Approximation for Phylogenetic Bootstrap. *Mol. Biol. Evol.* **30**, 1188–1195 (2013).

22. Guindon, S. et al. New algorithms and methods to estimate maximum-likelihood phylogenies: Assessing the performance of PhyML 3.0. *Syst. Biol.* **59**, 307–321 (2010).

23. Ronquist, F. et al. MrBayes 3.2: efficient Bayesian phylogenetic inference and model choice across a large model space. *Syst. Biol.* **61**, 539–542 (2012).

24. Rambaut, A., Suchard, M. A., Xie, D. & Drummond, A. J. Tracer v1.6, Available from http://tree.bio.ed.ac.uk/software/tracer/ (2013)

25. Shimodaira, H. An Approximately Unbiased Test of Phylogenetic Tree Selection. *Syst. Biol.* **51**, 492–508 (2002).

26. Strimmer, K. & Rambaut, A. Inferring confidence sets of possibly misspecified gene trees. *Proc. Biol. Sci.* **269**, 137–142 (2002).

27. Shimodaira, H. & Hasegawa, M. Multiple Comparisons of Log-Likelihoods with Applications to Phylogenetic Inference. *Mol. Biol. Evol.* **16**, 1114–1116 (1999).

28. Kishino, H. & Hasegawa, M. Evaluation of the maximum likelihood estimate of the evolutionary tree topologies from DNA sequence data, and the branching order in hominoidea. *J. Mol. Evol.* **29**, 170–179 (1989).

29. Salichos, L., Stamatakis, A. & Rokas, A. Novel information theory-based measures for quantifying incongruence among phylogenetic trees. *Mol. Biol. Evol.* **31**, 1261–1271 (2014).

30. Chernomor, O., von Haeseler, A. & Minh, B. Q. Terrace Aware Data Structure for Phylogenomic Inference from Supermatrices. *Syst. Biol.* **65**, 997–1008 (2016).

31. Kerou, M., Alves, R. J. E. & Schleper, C. Nitrososphaeria. in *Bergey's Manual of Systematics of Archaea and Bacteria* 1–8 (John Wiley & Sons, Inc., in association with Bergey's Manual Trust, 2016). doi:10.1002/9781118960608.cbm00055

32. Shi, X. et al. Effects of the Nitrification Inhibitor 3,4-Dimethylpyrazole Phosphate on Nitrification and Nitrifiers in Two Contrasting Agricultural Soils. *Appl. Environ. Microbiol.* **82**, 5236–5248 (2016).

33. Zhang, Y., Chen, L., Dai, T., Sun, R. & Wen, D. Ammonia manipulates the ammonia-oxidizing archaea and bacteria in the coastal sediment-water microcosms. *Appl. Microbiol. Biotechnol.* **99**, 6481–6491 (2015).

34. Zhao, J., Wang, B. & Jia, Z. Phylogenetically distinct phylotypes modulate nitrification in a paddy soil. *Appl. Environ. Microbiol.* **81**, 3218–3227 (2015).

35. Wang, B. et al. Differential contributions of ammonia oxidizers and nitrite oxidizers to nitrification in four paddy soils. *ISME J.* **9**, 1062–1075 (2015).

36. Wang, B. et al. Active ammonia oxidizers in an acidic soil are phylogenetically closely related to neutrophilic archaeon. *Appl. Environ. Microbiol.* **80**, 1684–1691 (2014).

37. Wu, Y. et al. Autotrophic growth of bacterial and archaeal ammonia oxidizers in freshwater sediment microcosms incubated at different temperatures. *Appl. Environ. Microbiol.* **79**, 3076–3084 (2013).

38. Lu, L. & Jia, Z. Urease gene-containing Archaea dominate autotrophic ammonia oxidation in two acid soils. *Environ. Microbiol.* **15**, 1795–1809 (2013).

39. Zhang, L.-M., Hu, H.-W., Shen, J.-P. & He, J.-Z. Ammonia-oxidizing archaea have more important role than ammonia-oxidizing bacteria in ammonia oxidation of strongly acidic soils. *ISME J.* **6**, 1032–1045 (2012).

40. Pratscher, J., Dumont, M. G. & Conrad, R. Ammonia oxidation coupled to CO2 fixation by archaea and bacteria in an agricultural soil. *Proc. Natl. Acad. Sci. U. S. A.* **108**, 4170–4175 (2011).

41. Xia, W. et al. Autotrophic growth of nitrifying community in an agricultural soil. *ISME J.* **5**, 1226–1236 (2011).

42. Zhang, L.-M. et al. Autotrophic ammonia oxidation by soil thaumarchaea. *Proc. Natl. Acad. Sci. U. S. A.* **107**, 17240–17245 (2010).

43. Jiang, X. et al. pH regulates key players of nitrification in paddy soils. *Soil Biol. Biochem.* **81**, 9–16 (2015).
