# Supplementary material for: Unifying the global phylogeny and environmental distribution of ammonia-oxidising archaea based on amoA genes
Source: Nat Commun. 2018 Apr 17;9:1517. doi: 10.1038/s41467-018-03861-1 (PMC5904100; doi:10.1038/s41467-018-03861-1)
Supplement: Supplementary file 3 — Description of Additional Supplementary Files [file 41467_2018_3861_MOESM3_ESM.pdf]

Description of Additional Supplementary Files

- Supplementary Data 1 – Reference alignments of archaeal *amoA* sequences
- Supplementary Data 2 – Reference phylogenetic trees of archaeal *amoA* genes
- Supplementary Data 3 – Reference databases for classification of archaeal *amoA* sequences with QIIME[1], mothur[2], LCAClassifier/CREST[3], MEGAN[4] and EPA[5]
- Supplementary Data 4-6 – Krona[6] interactive charts of AOA clade frequency *per* habitat and overall taxonomic distribution of archaeal *amoA* sequences
- Supplementary Data 7-9 – Krona[6] interactive charts of habitat frequency *per* AOA clade
- Supplementary Data 10 – Krona[6] interactive chart of molecular traits *per* AOA clade
- Supplementary Data 11 – GenBank file parsing script
- Supplementary Data Description References

Supplementary Data 1. Reference alignments of archaeal *amoA* sequences**.** Data contents:

AamoA.db_an96.aln_tax.annotated.fasta. Multiple sequence alignment of archaeal *amoA* OTU representative sequences in the curated database clustered at 96% identity (1,206 sequences) in FASTA format with taxonomically annotated labels.

AamoA.db_all.seqs.fasta. Full curated archaeal *amoA* sequence database (33,378 sequences) in FASTA format, unaligned and without taxonomic annotations.

AamoA_chimera.ref.db_aln.fasta. Reference database in FASTA format (411 aligned sequences) for chimera detection with UCHIME[7] and ChimeraSlayer[8] using the optimised parameters described in the Methods section. It is advisable to inspect the filtering results for potential false-positives resulting from novel diversity not covered by the database (*e.g.*, detection of an unusually large number of chimeras), and use less stringent parameters if necessary. Please also note that the database and parameters were optimised for UCHIME[7], and that the recent UCHIME2[9] version was designed for greater sensitivity using very large and comprehensive reference databases. Our preliminary tests indicated that using UCHIME2[9] with the current reference database and default parameters results in high false-positive detection rates. We recommend using *e.g.*, UCHIME[7] with the present database as a second filtering step, after filtering the new sequences/reads with a more sensitive *de novo* method that can profit from the sequence/read abundance information in the original dataset (*e.g.*, UPARSE[10]).

Supplementary Data 2. Reference phylogenetic trees of archaeal *amoA* genes**.** Data contents:

AamoA.db_tree.taxonomy_supported.nexus. Phylogenetic tree of archaeal *amoA* genes (shown in Fig. 1) in NEXUS format, with taxonomic annotations in sequence labels and clades, compatible with FigTree (http://beast.bio.ed.ac.uk/figtree). Only branches with both ultrafast bootstrap[11] ≥95% and SH-aLRT[12] ≥85% are shown, and other branches are represented as multifurcations (see legend of Fig. 1 for tree description). The taxonomic hierarchy represented in the tree can be navigated by collapsing/uncollapsing the respective clades; nodes/clades that do not correspond to taxonomic ranks do not display a taxonomic label when collapsed (see Methods and Results sections for taxonomic definitions). The tree is fully editable with FigTree and can be exported in different formats to prepare user-defined figures.

AamoA.db_tree.all.branches_labelled.newick. Phylogenetic tree of archaeal *amoA* genes in Newick format, with strictly-bifurcating branches and branch support values (before collapsing branches with low support; see Methods). Each branch contains two support values separated by "/", corresponding to SH-aLRT[12]/ultrafast bootstrap[11], which can be visualised with FigTree (http://beast.bio.ed.ac.uk/figtree).

Supplementary Data 3. Reference databases for classification of archaeal *amoA* gene sequences with QIIME[1], mothur[2], LCAClassifier/CREST[3], MEGAN[4] and EPA[5]. Data contents:

AamoA.db_nr_qiime.mothur. Reference database of archaeal *amoA* sequences in FASTA format for classification with QIIME[1] and mothur[2] using the taxonomy mapping files AamoA.db_nr.aln_taxonomy_qiime.txt and AamoA.db_nr.aln_taxonomy_mothur.txt, respectively. The sequence alignment includes all curated non-redundant *amoA* sequences in the database (*i.e.*, unique sequences). The database is compatible with all classification methods available in the *classify.seqs* command in mothur[2], and with all classification methods available in the *assign_taxonomy.py* command in QIIME[1], with the exception of the RDP classifier method, which

is restricted to six taxonomic levels and will likely omit several levels in the current taxonomy (which contains up to 11 levels).

AamoA.db_nr_lcaclassifier.megan_v0.7. Reference databases of archaeal *amoA* sequences for classification with the Lowest Common Ancestor algorithm (LCA) implemented in the LCAClassifier (CREST package)[3] and in MEGAN[4]. New sequences must be first aligned to the reference database (amoa.fasta) with BLASTN[13] using the binary search indices provided (amoa.fasta.nhr, amoa.fasta.nin and amoa.fasta.nsq), and the output files can then be used for classification with either the LCAClassifier[3] (amoA.map and amoa.tre) or MEGAN[4] (amoA_megan.map and amoA_megan.tre) using the respective mapping and tree files provided (indicated in brackets above). The archaeal *amoA* database is also automatically available in the current version of LCAClassifier[3] upon installation, without requiring external files. Software downloads and detailed usage instructions can be found at:
LCAClassifier/CREST[3]: http://apps.cbu.uib.no/crest/index
MEGAN[4]: http://ab.inf.uni-tuebingen.de/data/software/megan6/download/welcome.html

AamoA.db_an96.roguesout_epa. Phylogenetic tree of archaeal *amoA* genes in Newick format and corresponding multiple sequence alignment in FASTA format, compatible with the Evolutionary Placement Algorithm (EPA) implemented in RAxML[5]. The tree has strictly-bifurcating branches but no branch support values, as RAxML does not support the original branch support value format. The sequence alignment includes all OTU representatives of the curated database clustered at 96% identity, excluding rogue sequences (1,990 sequences; see Methods).

NOTE: Supplementary Data 4-10 are interactive Krona[6] charts in HTML format, which can be navigated with an internet browser, as described below.

Supplementary Data 4-6. Krona[6] interactive charts of AOA clade frequency *per* habitat. Charts display the absolute and relative taxonomic distribution of *amoA* sequences in the curated database, on every taxonomic level, originating from five broad habitats and specific niches therein. The different habitat categories and subcategories can be selected from the lists in the top-left corner and the taxonomy can be browsed by clicking on the chart wedges; absolute and relative sequence abundances *per* taxon are shown in the top-right corner of the page and on the chart, respectively. Habitat categories are divided between charts as follows:

Supplementary Data 4. AOA clade frequency *per* habitat (categories 1). Marine, estuarine-coastal, soils-sediments, freshwater and salt lakes. The taxonomic distribution of all sequences irrespective of environmental source is displayed under section "All sequences".

Supplementary Data 5. AOA clade frequency *per* habitat (categories 2). Marine habitats: water, sediments, eukaryote-associated and aquaria; estuarine-coastal habitats: estuaries-bays and marshes-mangroves; freshwater habitats: water-sediments, subsurface water-sediments, hot springs, aquaria and wastewater treatment plants.

Supplementary Data 6. AOA clade frequency *per* habitat (categories 3). Marine habitats: ocean water depth, hydrothermal vent field sediments/precipitates and eukaryote-associated putative hosts; soils-sediments pH range.

Supplementary Data 7-9. Krona[6] interactive charts of habitat frequency *per* AOA clade (*i.e.*, habitat specificity). Charts display the fraction of archaeal *amoA* sequences in the curated database, on every taxonomic level, originating from five broad habitats and specific niches therein. Fractions were calculated in relation to total number of sequences assigned to a niche/habitat within the same

category level, excluding those only assigned to the broader category above (*e.g.*, the fraction of sequences from each seawater depth was calculated in relation to all sequences assigned to a specific depth, excluding those without depth information). The different habitat categories and subcategories can be selected from the lists in the top-left corner and the taxonomy can be browsed by clicking on the chart wedges; absolute and relative sequence abundances per taxon are shown in the top-right corner of the page and on the chart, respectively. The option "Color by % of assigned sequences in clade" (below the habitat list) must be selected to visualise the fraction of sequences in each clade according to the colour gradient in the bottom-left corner. Description of calculations and value interpretations are provided in the Methods section. Habitat categories are divided between charts as follows:

Supplementary Data 7. Habitat frequency *per* AOA clade (categories 1). Marine, estuarine-coastal, soils-sediments, freshwater and salt lakes.

Supplementary Data 8. Habitat frequency *per* AOA clade (categories 2). Marine habitats: water, sediments, eukaryote-associated and aquaria; estuarine-coastal habitats: estuaries-bays and marshes-mangroves; freshwater habitats: water-sediments, subsurface water-sediments, hot springs, aquaria and wastewater treatment plants.

Supplementary Data 9. Habitat frequency *per* AOA clade (categories 3). Marine habitats: water depth, hydrothermal vent field sediments/precipitates and eukaryote-associated putative hosts; soils-sediments pH range.

Supplementary Data 10. Krona[6] interactive chart of molecular traits *per* AOA clade. Chart of taxonomic distribution of variation in DNA base composition and synonymous codon usage of archaeal *amoA* genes on every taxonomic level. The charts display the fraction of maximum positive and negative deviation in GC %, purine %, Nc and gCAI of *amoA* genes in each clade, in relation to their respective global averages. gCAI values represent the fraction of maximum variation in deviation from the dominant codon usage bias among all *amoA* genes. See Fig. 8 and Methods section for calculations and value interpretation. The different molecular features can be selected from the lists in the top-left corner and the taxonomy can be browsed by clicking on the chart wedges; fractions per taxon and number of sequences considered are shown in the top-right corner of the page and on the chart, respectively. The option "Color by % of maximum deviation" (below the feature list) must be selected to visualise the fraction of the maximum deviation in each clade, according to the colour gradient in the bottom-left corner.
*To avoid obscuring colour gradient patterns due to abnormally low Nc and gCAI of genes in three particular OTUs (see long branch within clade NP-θ in Fig. 8c, d), fractions of maximum negative deviation in Nc and gCAI were calculated in relation to the second lowest deviation values.

Supplementary Data 11. GenBank file parsing script. Python 2.7 script that extracts specified annotation information from GenBank files (.gb) as tab-formatted text data. By default, the following information is extracted, if present in the file: Name (*i.e.*, accession number), Source, Organism, Title, Journal, isolation_source, country, lat_lon and PCR_primers.

Supplementary Data Description References

1. Caporaso, J. G. *et al*. QIIME allows analysis of high-throughput community sequencing data. *Nat. Methods* **7,** 335–336 (2010).

2. Schloss, P. D. et al. Introducing mothur: open-source, platform-independent, community-supported software for describing and comparing microbial communities. *Appl. Environ. Microbiol.* **75**, 7537–7541 (2009).

3. Lanzén, A. *et al.* CREST – Classification Resources for Environmental Sequence Tags. *PLoS One* **7,** e49334 (2012).

4. Huson, D. H., Mitra, S., Ruscheweyh, H.-J., Weber, N. & Schuster, S. C. Integrative analysis of environmental sequences using MEGAN4. *Genome Res.* **21,** 1552–1560 (2011).

5. Berger, S. A., Krompass, D. & Stamatakis, A. Performance, accuracy, and web server for evolutionary placement of short sequence reads under maximum likelihood. *Syst. Biol.* **60**, 291–302 (2011).

6. Ondov, B. D., Bergman, N. H. & Phillippy, A. M. Interactive metagenomic visualization in a Web browser. *BMC Bioinformatics* **12**, 385 (2011).

7. Edgar, R. C., Haas, B. J., Clemente, J. C., Quince, C. & Knight, R. UCHIME improves sensitivity and speed of chimera detection. *Bioinformatics* **27**, 2194–2200 (2011).

8. Haas, B. J. et al. Chimeric 16S rRNA sequence formation and detection in Sanger and 454-pyrosequenced PCR amplicons. *Genome Res.* **21**, 494–504 (2011).

9. Edgar, R. C. UCHIME2: improved chimera prediction for amplicon sequencing. bioRxiv 74252 (2016). doi:10.1101/074252

10. Edgar, R. C. UPARSE: highly accurate OTU sequences from microbial amplicon reads. *Nat. Methods* **10,** 996–998 (2013).

11. Minh, B. Q., Nguyen, M. A. T. & von Haeseler, A. Ultrafast Approximation for Phylogenetic Bootstrap. *Mol. Biol. Evol.* **30**, 1188–1195 (2013).

12. Guindon, S. et al. New algorithms and methods to estimate maximum-likelihood phylogenies: Assessing the performance of PhyML 3.0. *Syst. Biol.* **59**, 307–321 (2010).

13. Altschul, S. F. et al. Gapped BLAST and PSI-BLAST: A new generation of protein database search programs. *Nucleic Acids Res.* **25**, 3389–3402 (1997).
